# Vegetarian diet duration’s influence on women’s gut environment

**DOI:** 10.1186/s12263-021-00697-1

**Published:** 2021-10-02

**Authors:** Xinqi Deng, Jiangtao Si, Yonglong Qu, Li Jie, Yuansong He, Chunguo Wang, Yuping Zhang

**Affiliations:** 1 School of Life Science, Beijing University of Chinese Medicine, Beijing, China; 2 Special Treatment Center, Wang Jing Hospital of Chinese Academy of Traditional Chinese Medicine, Beijing, China; 3 School of Traditional Chinese Medicine, Beijing University of Chinese Medicine, Beijing, China; 4 Sichuan Vocational College of Nursing, Chengdu, China; 5 Beijing Research Institute of Chinese Medicine, Beijing University of Chinese Medicine, Beijing, China

**Keywords:** Vegetarian diet, Gut microbiota, Fecal metabolites, Female

## Abstract

**Background:**

Nutrient composition of vegetarian diets is greatly different from that of omnivore diets, which may fundamentally influence the gut microbiota and fecal metabolites. The interactions between diet pattern and gut environment need further illustration. This study aims to compare the difference in the gut microbiota and fecal metabolites between vegetarian and omnivore female adults and explore associations between dietary choices/duration and gut environment changes.

**Methods:**

In this study, investigations on the fecal metabolome together with the gut microbiome were performed to describe potential interactions with quantitative functional annotation. In order to eliminate the differences brought by factors of gender and living environment, 80 female adults aged 20 to 48 were recruited in the universities in Beijing, China. Quantitative Insights Into Microbial Ecology (QIIME) analysis and Ingenuity Pathway Analysis (IPA) were applied to screen differential data between groups from gut microbiota and fecal metabolites. Furthermore, weighted gene correlation network analysis (WGCNA) was employed as the bioinformatics analysis tool for describing the correlations between gut microbiota and fecal metabolites. Moreover, participants were further subdivided by the vegetarian diet duration for analysis.

**Results:**

GPCR-mediated integration of enteroendocrine signaling was predicted to be one of the regulatory mechanisms of the vegetarian diet. Intriguingly, changes in the gut environment which occurred along with the vegetarian diet showed attenuated trend as the duration increased. A similar trend of returning to “baseline” after a 10-year vegetarian diet was detected in both gut microbiota and fecal metabolome.

**Conclusions:**

The vegetarian diet is beneficial more than harmful to women. Gut microbiota play roles in the ability of the human body to adapt to external changes.

**Supplementary Information:**

The online version contains supplementary material available at 10.1186/s12263-021-00697-1.

## Contributions to the literature

- A vegetarian diet could change women’s gut environment.
- GPCR-mediated integration of enteroendocrine signaling might be one of the regulatory mechanisms.
- Different diet choices exerted a significant impact on the gut environment within 5-10 years’ duration and then inversion happened in the following years.
- Gut microbiota and fecal metabolites interacted and changed in a synergistic manner in the human body, which helps to adjust to different dietary choices.

## Introduction

There is no single type of vegetarian diet. Instead, a vegetarian diet usually consists of three types: vegan, which excludes all meat and animal products; lacto vegetarian, which includes plant foods plus dairy products; and lacto-ovo-vegetarian, which includes both dairy products and eggs. By geography, European and North American vegetarians are mostly lacto-ovo-vegetarians [1], while lacto-vegetarians account for most part of Asian and Indian vegetarians [2, 3]. The definition of vegetarian diet also varies among studies [2, 4–8]. Consuming various kinds of food, especially the ones rich in protein, iron, calcium, zinc, and vitamin B12, is recommended for an appropriately planned vegetarian diet [9]. The diet pattern is believed to have a great impact on human health. However, whether the vegetarian diet is good for health remains controversial and how it affects the gut environment is still unclear. Some studies show that diet enriched with fruit, vegetables, and fiber leads to a lower risk of coronary diseases, hypertension, cancer, and type 2 diabetes [10–13]. But other studies suggest adverse effects brought by the vegetarian diet, including a higher risk of cardiovascular disease, osteoporosis, and malnutrition [14–17].

Dietary fiber, carbohydrates, fat, and protein are well-known nutrients in the modulation of the gut microbiota [18–22]. Gut microbiota refers to a huge microbial community which consists of more than 100 trillion bacteria, archaea, fungi, and protozoa. These commensal microorganisms are believed to play roles in maintaining the physiology of the host [23, 24] and regulate various conditions including inflammatory bowel disease [25–27], obesity [28], diabetes [29], autism spectrum disorder [30], and cardiovascular disease [31, 32]. Gut microbiota can be influenced and shaped by long-term diet [33–37] and impacts on human health via multiple metabolites [38] in turn. Therefore, the composition and metabolic products of gut microbes together constitute the gut environment, a larger concept for discussion. Differences in microbiota compositions between vegetarians and omnivores had arisen scientists’ interest and were discussed by previous studies [39–41]. However, the changing process of the gut environment along with different diet durations remains unclear, and it needs further study. Do composition and metabolic products of gut microbes keep a single trend in response to diet duration? To address this question, we investigated the gut environment changes under different diet types/durations. The vegetarian diet was reported to play roles in hormone flux, which is greatly related to women’s health [42–47]. Therefore, we focus on the relationship between the vegetarian diet duration and the women’s gut environment. In order to give comprehensive investigations, we profiled both metabolic activity in the gut by fecal metabolomics, and gut microbiota shaping by 16S ribosomal DNA sequencing. This study screened out gut microbiota and fecal metabolites for characterizing the omnivore and vegetarian diet as well as the vegetarian diet duration in women, trying to construct the potential interaction network between gut microbiota and fecal metabolites. The results described an interesting and consistent expression trend returning to “baseline” as the vegetarian diet duration prolonged to > 10 years, in both gut microbiota and fecal metabolites. As far as we know, this is the first report about the returning trend in the vegetarian gut environment, which may provide a reference for further study in diet, microbiota, and health.

## Result

### Fecal metabolic shifts regarding different dietary choices

The cross section [48] (Table 1) consists of 80 healthy females, including 46 vegetarians (vegan, ovo-vegetarian, and lacto-ovo-vegetarian diet) and 34 omnivores. All participants are subjected to a similar living environment and activity schedule in university. No significant difference exists between the vegetarian and omnivore groups in age and BMI.

**Table 1:** Groups of the vegetarian and the omnivore

Characteristics	Vegetarian group (*n* = 46)	Omnivore group (*n* = 34)	*Z*	*p*
Age, years				
Median	29	25	−1.84	0.07
Range	20-48	21-42		
BMI, kg/m^2^				
Median	22.4	22.0	−1.44	0.15
Range	17.6-46.9	15.1-48.1		

Data of age and BMI was analyzed with the binomial test

**Fig. 1:**
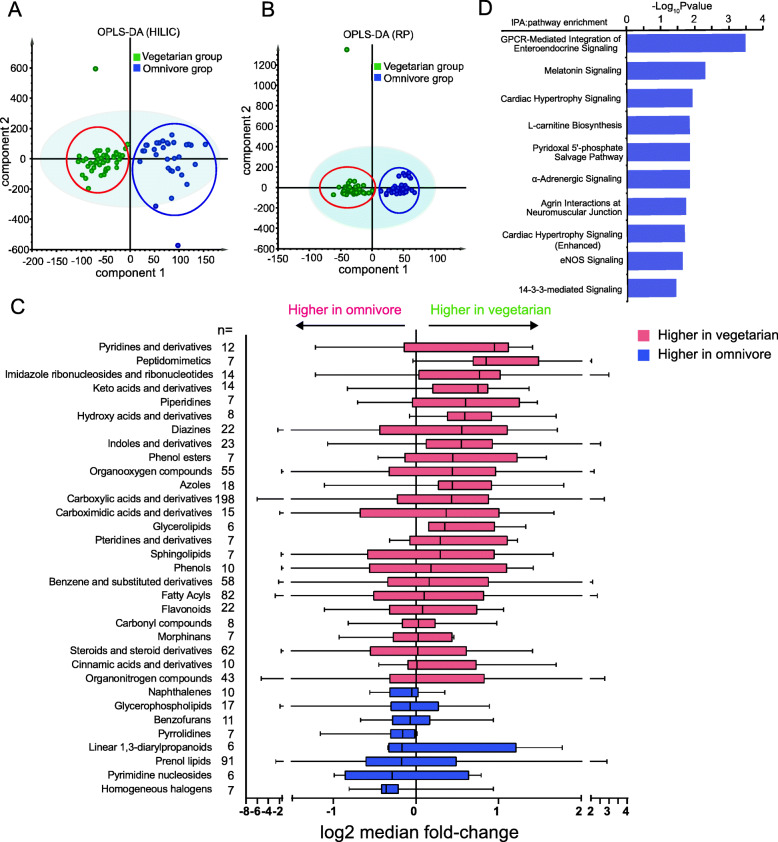
Fecal metabolic study regarding different diet choices. **A**, **B** The difference between the vegetarian and omnivore groups was illustrated with OPLS-DA results based on data under RP-C18 and HILIC modes (R2X = 0.62, R2Y > 0.6, Q2 > 0.6). **C** Metabolic enrichments in the vegetarian versus omnivore groups. Individual differential abundance trends were calculated with log2 fold change of vegetarian versus omnivore, focusing on classes of molecules with at least 5 putative members (see the “*n*=”column). Classes of molecules were identified according to the HMBD database. **D** Major pathways enriched with IPA

A set of differential fecal metabolites between the vegetarian and omnivore groups were screened by employing ANOVA and orthogonal projections to latent structures discriminant analysis (OPLS-DA). The results are illustrated by volcano plots (Figure S1A, B) and OPLS-DA (Fig. 1A, B, Figure S1C, D). Ion fragments were screened for chemical structure identification on the basis of automatic mapping analysis result, followed by manual analysis for further quality control. Six hundred forty-eight compounds were assigned into 33 classes based on HMDB annotations with at least 5 members. Twenty-five of the 33 molecular classes showed higher abundance in vegetarian participants, among which pyridines and derivatives, peptidomimetics, and imidazole ribonucleosides and ribonucleotides displayed the strongest shifts (Fig. 1C). Differential metabolites were screened with both OPLS-DA (VIP > 1) and one-way ANOVA (*p* < 0.05, |fold change|> 1.2). After that, 70 endogenous compounds (Table S1) were identified for further bioinformatics analysis. Potential pathways were enriched with IPA (Fig. 1D), which mainly involved in two aspects: GPCR-mediated enteroendocrine signalings including melatonin signaling, l-carnitine biosynthesis, pyridoxal 5′-phosphate salvage pathway, and α-adrenergic signaling; and cardiovascular functions related to cardiac hypertrophy, agrin interactions, and eNOS signaling.

### Effect of dietary choice on the gut microbiome composition

**Fig. 2:**
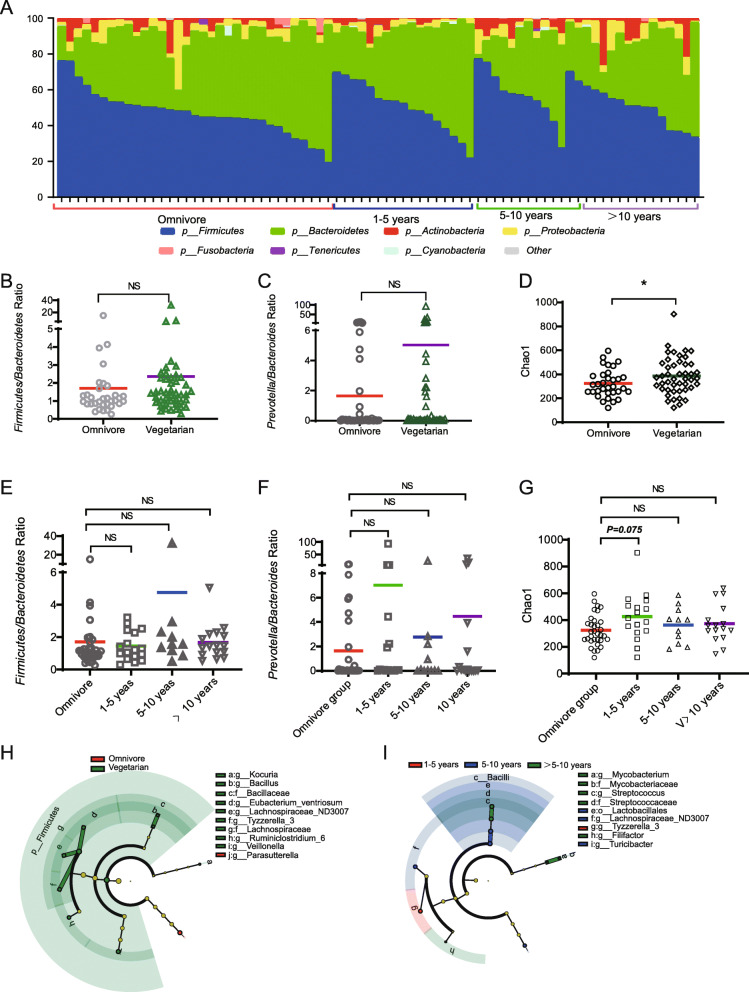
**A** Distribution of predominant gut bacterial phylotypes in participants under omnivore and 1-5 years, 5-10 years, and > 10 years of vegetarian diet duration. **B**, **E** Firmicutes/Bacteroidetes ratio of different groups, *t* test. **C**, **F** Prevotella/Bacteroidetes ratio of different groups, *t* test. **D**, **G** Species richness (Chao1 index) of different groups, *t* test, *p < 0.05. **H**, **I** Gut microbes which play roles in the distinction of different groups

In the gut microbiome study, 16S ribosomal DNA sequencing assay was employed for the identification and relative abundance detection of gut bacterial populations. By merging all detected bacteria at the taxonomic level of class, family, genus, order, and phylum, 447 gut microbes, which were common in both vegetarian and omnivore groups, were screened and identified. Matching well with a previous report [49], the dominant bacterial phyla in the global participants were *Firmicutes* and *Bacteroidetes*, with smaller proportions of *Proteobacteria* and *Actinobacteria* (Fig. 2A). *Firmicutes/Bacteroidetes* ratio was reported as a possible biomarker of gut environment changes in many cases including gallstone disease [50], obesity [51], inflammatory bowel disease [52], and aging [53]. The *Firmicutes*/*Bacteroidetes* ratio was calculated, but no significant difference was shown between the vegetarian and omnivore groups (Fig. 2B). *Prevotella*-to-*Bacteroides* ratio was reported as an indicator of body weight and fat loss [54, 55]. According to our data, *Prevotella/Bacteroides* ratio showed no significant difference between the vegetarian and omnivore groups (Fig. 2C). By performing alpha diversity analysis, the Chao1 estimator was applied for gut microbiota comparison between the vegetarian and omnivore groups, and a statistically significant difference was shown. The vegetarian group presented a higher community richness in the gut microbiota (Fig. 2D). In this context, it was indicated that the vegetarian diet choice does not exert a significant negative impact on women’s health. Moreover, significant differences in the gut microbial composition between the vegetarian and omnivore groups were identified with LEfSe analysis. LEfSe analysis result would help to identify the taxonomic groups which are relevant in characterizing each dietary habit. Accordingly, 8 genera and 2 families, including *Kocuria*, *Bacillus*, *Bacillaceae*, *Ruminiclostridium*, *Lachnospiraceae*, *Eubacterium*, *Tyzzerella*, *Veillonella*, *Parasutterella*, and *Lachnospiraceae*, were found as potential factors that make differences between the vegetarian and omnivore groups (Fig. 2H).

### Major gut environment shaping by vegetarian diet duration

**Fig. 3:**
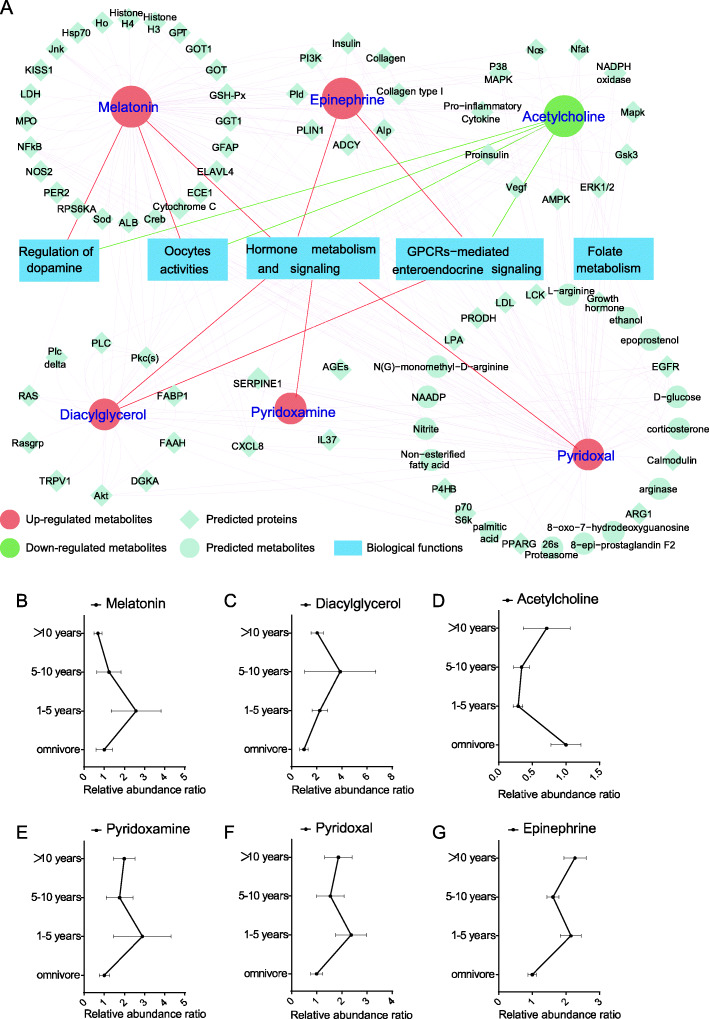
**A** Network analysis result. Metabolites upregulated in the vegetarian group were marked in red, and the downregulated ones were marked in green. **B**-**G** Relative abundance ratio (vegetarian group/omnivore group) of 6 metabolites being involved in the top 3 pathways. Data is presented as mean ± *SEM*, *t* test

In order to investigate the relationship of vegetarian diet duration and gut environment, the vegetarian group was divided into three sub-groups according to the length of vegetarian diet. No bias occurred in participant characteristics in groups of different vegetarian diet durations (Table 2). Based on metabolites which engage in the top 3 pathways (Fig. 1D), a network analysis was performed with IPA. Items are allowed to connect to others directly or indirectly (via third party) to build up an integrated network in IPA biological function network construction. In this study, a GPCR-mediated enteroendocrine regulation network was constructed (Fig. 3A) with 6 fecal metabolites, including pyridoxal, diacylglycerol, acetylcholine, pyridoxamine, epinephrine, and melatonin. Relative abundance ratio (vegetarian group/omnivore group) was compared (Fig. 3B-G). Interestingly, melatonin (Fig. 3B), diacylglycerol (Fig. 3C), and acetylcholine (Fig. 3D) showed dramatic changes within 10 years of vegetarian diet duration and then inversion happened in the following years. Pyridoxamine (Fig. 3E), pyridoxal (Fig. 3F), and epinephrine (Fig. 3G) showed dramatic changes within 5 years of vegetarian diet duration followed by two inversions.

**Table 2:** Groups of different vegetarian diet duration

Characteristics	Omnivore (*n* = 34)	1-5 years (*n* = 16)	5-10 years (*n* = 16)	> 10 years (*n* = 14)	*Z*	*p*
Age, years						
Median	26	28	26	30	0.540	0.910
Range	22-43	20-38	20-48	20-41		
BMI, kg/m^2^						
Median	20.9	21.8	20.5	22.2	1.693	0.639
Range	15.1-48.1	17.6-46.9	18.0-27.7	18.4-24.8		

Data of age and BMI was analyzed with the Kruskal algorithm

**Fig. 4:**
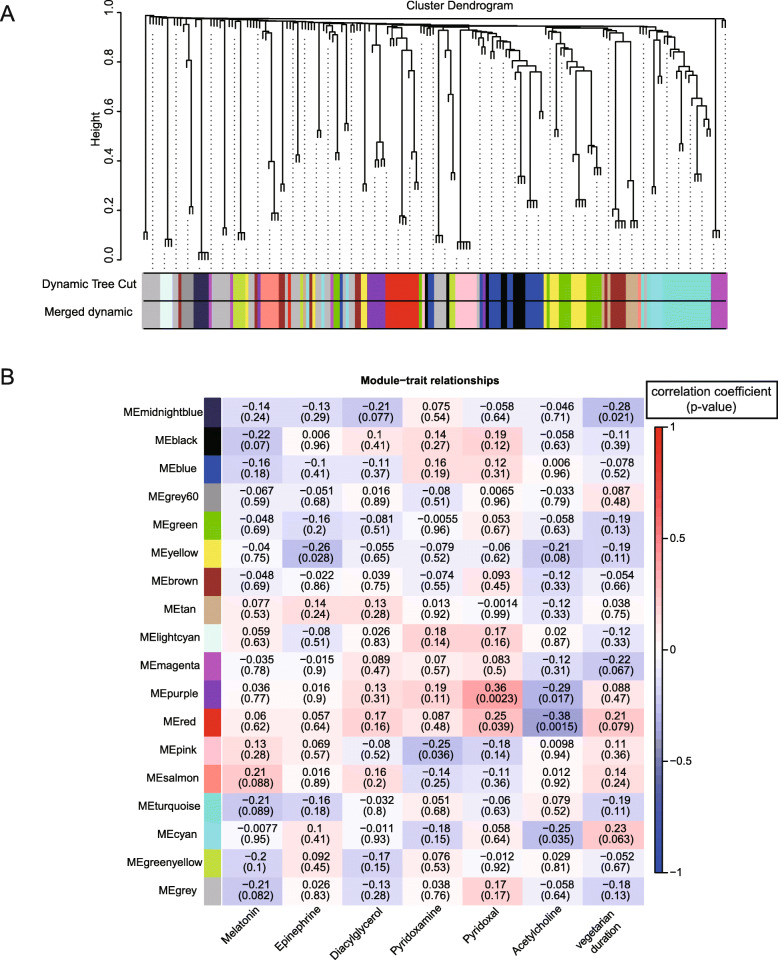
WGCNA based on 447 common gut microbes identified in each sample. **A** Eighteen co-occurring gut microbe modules obtained based on the data set of 447 microbes. **B** Correlation coefficient matrix presenting module-trait relationships based on the Pearson method, and correlation coefficient were marked on each square with *p* value presented in brackets. Factors were supposed to be correlated with |correlation coefficient|> 0.25 and *p* value < 0.05

**Fig. 5:**
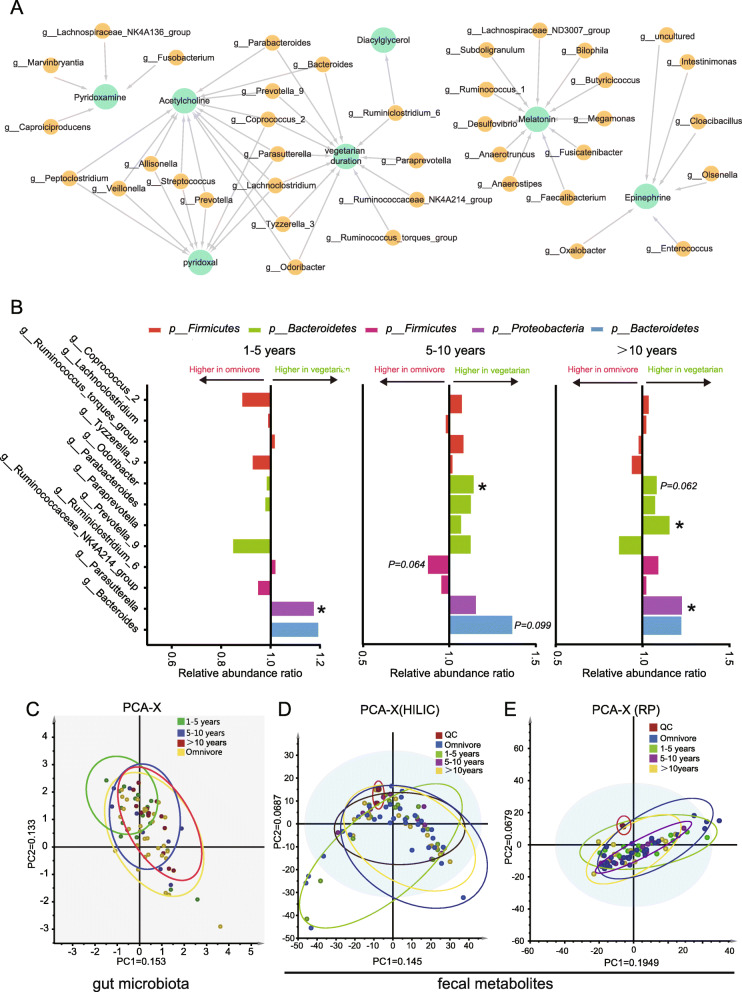
**A** Co-occurrence relationships with Spearman’s correlation coefficient that *p* < 0.1 are depicted with network diagram for traits including key metabolites and the vegetarian diet duration. **B** Changes in the abundance of the vegetarian diet duration-associated microbial genera as the vegetarian diet duration prolonged (*t* test, *p < 0.05). **C**-**E** PCA describing migrations of the gut microbiome (**C**) and fecal metabolism data (**D**, **E**) along with the vegetarian diet duration

Next, the influence of vegetarian diet duration on gut microbiota shaping was looked into. *Firmicutes/Bacteroidetes* ratio was compared among the groups of 1-5 years, 5-10 years, and > 10 years of vegetarian diet. It was found that participants under 5-10 years of vegetarian diet had the highest *Firmicutes/Bacteroidetes* ratio (Fig. 2E). And participants under 5-10 years of vegetarian diet were found to have the lowest *Prevotella/Bacteroides* ratio (Fig. 2F), which indicates body weight and fat loss induced by the vegetarian diet to some extent. Interestingly, the return to “baseline” after 10 years of vegetarian diet was shown again in *Prevotella/Bacteroides* ratio (Fig. 2F), just like the one of *Firmicutes/Bacteroidetes* ratio (Fig. 2E). Though the Chao1 estimator failed to show statistically significant differences in community richness among the three groups (Fig. 2G), 6 genera, 2 families, and 1 order of microorganisms were found accountable for the distinctiveness of groups (Fig. 2I). Taken together, *Lachnospiraceae_ND3007_group* and *Tyzzerella_3* were identified to be responsible for the distinction in both different dietary choices and durations. By employing WGCNA, 18 co-occurring gut microbe modules were obtained based on the data set of 447 microbes (Fig. 4A). One hundred ninety-three microbes were grouped in co-occurring gut microbe modules (Table S2). A total of 12 co-occurring microbial genera were screened to be possibly correlated with the vegetarian diet duration with |correlation coefficient|> 0.25 and *p* value < 0.05 (Figs. 4B and 5A, Table S3). By comparing the abundance of these 12 microbial genera among the groups of different vegetarian diet durations, it was shown that most of them achieved a higher abundance ratio of vegetarian group/omnivore group upon 5-10 years of vegetarian diet, which then was neutralized or even reversed as the vegetarian diet duration prolonged. These results show a pattern similar to some of the gut metabolites (Fig. 5B).

Furthermore, by performing unsupervised PCA, we explored how the gut environment changed as a whole in response to different vegetarian diet duration. For PCA, distance along the *X*-axis illustrates the distinction among the groups. For the gut microbial genera data, it was shown that the omnivore group position on the right, while the 1-5 years vegetarian group was positioned on the far left away from the omnivore circle. As the vegetarian diet duration prolonged, the position of PCA circles moved toward the omnivore group (Fig. 5C). In order to obtain wider coverage of the metabolome polarity range, both HILIC- (Fig. 5D) and RP- (Fig. 5E) liquid chromatography separations were applied for metabolomics data collection. The HILIC data displayed a similar variation pattern as that of the gut microbial genera data: the 1-5 years vegetarian group showed the greatest distinction from the omnivore group, and the differences from the omnivore group were minimized as the vegetarian diet duration prolonged. However, the non-obvious distinction was shown under the RP-separation mode. Taken together, the PCA results displayed synergistic variations between gut microbiota genera and fecal metabolites. The 1-5 years of vegetarian diet duration caused drastic changes in women’s gut environment. As the vegetarian diet duration prolonged, the variation became weaker and displayed less difference than the omnivores.

## Discussion

Gut microbiota refers to a complex community of commensal bacteria mostly influenced by dietary nutrients [56]. It plays a fundamental role in host physiology [24, 57, 58]. Gut microbes interact with the host via various metabolites which can be reabsorbed by the host. In turn, variations in fecal metabolites are likely to be caused by a combination of factors including diet patterns, host metabolites, and modified microbiota-host interactions. Theoretically, more fiber, less fat, and different types of protein make the vegetarian diet shape a different gut environment from that of omnivore. Though some studies highlighted the difference in the gut microbiota between vegetarians and omnivores [59], contradictory or non-significant results were reported in other studies [60]. Moreover, since an intervention study can only catch some short-term or reversible changes in the gut environment, investigations are needed in the long-term effects of diet patterns on gut microbiota and fecal metabolites, especially by comparing data from the groups of different vegetarian diet durations.

In this study, a good separation between the vegetarian and omnivore groups was shown in metabolomics data with OPLS-DA analysis. Significant differences in the gut microbiota were found between omnivore and vegetarian populations. These results suggest that different diet patterns can lead to significant shifts in gut microbiota and fecal metabolites in the host. By processing differential metabolic data with the IPA software, GPCR-mediated integration of enteroendocrine signaling was enriched with the highest score. G protein-coupled receptors (GPCRs) are believed to be key mediators in the interactions of gut microbes and host physiology [61]. The enteroendocrine system functions as a sensor of the intestinal components and body energy status and reacts to ingested food relying on GPCRs. Moreover, as the largest endocrine system in the body, it exerts regulations on hormone secretion and interacts with the host physiological system [62]. Previous research showed that the concentration and activity of sex hormones, especially estrogens, can be strongly influenced by the vegetarian diet [42–47]. Also, the vegetarian diet was found to increase fecal estrogen excretion [46]. Several hormone secretion-related pathways were enriched based on differential fecal metabolites, including pyridoxal, pyridoxamine, diacylglycerol, epinephrine, and melatonin, in this study. Vitamin B6 had been reported to play roles in the secretion and function of various hormones [63]. Pyridoxal and pyridoxamine, two major members of the vitamin B6 group, had been reported to have a great impact on women’s health [64–68]. With upregulated vitamin B6 group, the vegetarian diet was suggested to enhance the ability to withstand disease risks including coronary heart disease [69], colorectal cancer [70], breast cancer [71], and ovarian cancer [72] in women. Intriguingly, vitamin B6 also plays key roles in the metabolism of hormones such as melatonin and epinephrine [73]. Melatonin is a chemical which helps people establish a day and night cycle and corrects sleep disorders. Also, as important chemical messengers, melatonin and epinephrine exert regulations on a set of reproductive actions including ovarian follicle growth, ovulation, luteinization, fertilization, implantation, pregnancy, and parturition via the hypothalamus–pituitary–gonadal axis [74–78]. Besides, l-carnitine plays a key role in shaping health conditions. l-carnitine functions by carrying fatty acids across the mitochondrial membrane for beta-oxidation [79] and can be converted into TMAO via gut microbiota and host enzymes [80]. A carnitine-rich diet was associated with incident risks of myocardial infarction, stroke, and death, significantly in patients with concomitantly high TMAO levels in plasma [81]. However, a clear mechanistic link between TMAO and such diseases is not yet validated. Whether increased TMAO concentrations are the cause or result of these, diseases remained controversial [82]. It was reported that a transient switch to the vegetarian diet reduced body creatine pool without affecting the carnitine homeostasis [83]. In this study, there was no significant difference in fecal l-carnitine level between the vegetarians and e omnivore group, which is consistent with the previous report [83]. Taken together, the vegetarian diet is beneficial more than harmful for women’s health and quality of life.

In addition, changes in gut microbiota and fecal metabolites were illustrated upon different vegetarian diet duration from multiple perspectives, and consistent conclusions were drawn. The differences between the vegetarian and omnivore groups in both fecal metabolites and gut microbiota became weaker over the length of the vegetarian diet duration. This suggests that gut microbiota and fecal metabolites interact and change in a synergistic manner in the human body, which helps to adjust to different dietary choices. It was demonstrated through metagenomic and biochemical analyses that changes in the relative abundance of the Bacteroidetes and Firmicutes affect the metabolic potential of the mouse gut microbiota [84]. We compared the Firmicutes/Bacteroidetes ratio among the groups for investigating the changes brought by different diets as well as the vegetarian diet duration. Besides, we have investigated the Prevotella/Bacteroides ratio of the groups in the revision and found the trend of returning to “baseline” after 10 years of vegetarian diet, which was similar to that of the Firmicutes/Bacteroidetes ratio. This provided another evidence for our conclusion that changes in the gut environment between omnivores and vegetarians weaken over the vegetarian diet duration.

Though participants were recruited within a similar living environment, types of vegetarianism are not discussed in the analyses. Short-chain fatty acids (SCFA) are very important components in studies on the gut environment, which can be detected by GC-MS rather than UPLC-MS. In this study, GC-MS was not employed; therefore, SCFA was not detected and reported. These are the limitations of this study. Nonetheless, UPLC-MS is perfect for detecting most of the components. In this study, both HILIC- and RP-liquid chromatography separation were applied for metabolomics data collection, which could be helpful in obtaining wider coverage of the metabolome polarity range.

## Conclusions

In conclusion, the vegetarian diet was found to change the expression of fecal metabolites and may influence the host via GPCR-mediated enteroendocrine regulation. Based on the similar trend of returning to “baseline” after 10 years of vegetarian diet in both gut microbiota and fecal metabolome, we deduced that distinctions in the gut environment between omnivores and vegetarians weaken over the vegetarian diet duration. This suggests that gut microbiota play roles in the ability of the human body to adapt to external changes.

## Methods

### Participants and grouping

Eighty healthy female individuals aged 20–48 years were recruited from universities in Beijing, China, in this study. Participants reported a similar living environment and similar activity schedule, including accommodation; air pollution; green space exposure; access to transit, primary care, facilities, and meals; course and recreation hours, which help improve assessment of balance in baseline characteristics. Participants under antibiotics, probiotics at the time of enrolment, and the ones diagnosed with serious diseases (cardiovascular, liver, kidney, hematopoietic dysfunction, etc.), mental disorders (epilepsy, depression, etc.), endocrine diseases (polycystic ovary syndrome, thyroid disease, diabetes, etc.), or gut issues (IBS, IBD, etc.) were excluded from the study. Participants following vegetarian/omnivore diet for less than 1 year were excluded, too. In this study, the omnivore group (*n* = 34) was defined as consuming a general diet; the vegetarian group (*n* = 46) consisted out of three sub-types: vegan, ovo-vegetarian, and lacto-ovo-vegetarian. Vegans consume plant foods such as fruits, vegetables, cereals, legumes, nuts, and seeds. Ovo-vegetarians consume plant foods and eggs. Lacto-ovo-vegetarians consume plant foods, as well as eggs and dairy products. Further, the vegetarian group was divided into three groups according to the length of the vegetarian diet: 1-5 years, 5-10 years, and > 10 years to investigate the relationship of the vegetarian diet duration and the gut environment.

### Questionnaire and statistical analysis

After signing the informed consent, each individual was asked to complete a questionnaire to provide information regarding their diet type, vegetarian diet duration, age, body mass index (BMI), and medical history. Participants were asked to describe their diet type with vegan (under diet based on plant foods only, including fruits, vegetables, cereals, legumes, nuts, and seeds), ovo-vegetarian (under diet based on plant foods and eggs), or lacto-ovo-vegetarians (under diet based on plant foods, eggs, and dairy products). The data were examined in the SPSS 22.0 software to calculate the statistical significance. Data of age and BMI were analyzed with the binomial test or Kruskal algorithm.

### Samples and microbiome analyses

Stool samples were collected once during the non-menstrual phase to avoid contamination. Samples were taken by the participants with tubes and then stored in DNA stabilizer solution at the − 80 °C refrigerators of the laboratory. Samples were transported in liquid nitrogen for further experiments after the collection was finished. A 0.2-g sample was milled in 1 ml methanol with bead mill (Retsch PM 400-MA, Germany). Supernatants were collected and stored at − 80 °C after centrifuged at 13000 rpm•min^−1^ for 20 min at 4 °C. DNA extraction was applied with PowerSoil DNA Isolation Kit (MoBio Laboratories, Carlsbad, CA) following the instructions. After checking the purity and quality of the genomic DNA with 1% agarose gels and a NanoDrop spectrophotometer (Thermo Scientific), 16S ribosomal DNA sequencing was performed. The V3-4 hypervariable region of bacterial 16S ribosomal DNA was amplified with the primers 338F (5′-ACTCCTACGGGAGGCAGCAG-3′) and 806R (5′-GGACTACNNGGGTATCTAAT-3′) [85]. For sample distinguishment, an 8-digit barcode sequence was added to the 5′ end of the forward and reverse primers (provided by Allwegene Company, Beijing). Conditions of PCR were as follows: Mastercycler Gradient (Eppendorf, Germany); reaction volumes (25 μl) containing 12.5 μl KAPA 2G Robust Hot Start Ready Mix, 1 μl forward primer (5 μM), 1 μl reverse primer (5 μM), 5 μl DNA (total template quantity is 30 ng), and 5.5 μl H_2_O; cycling parameters (95 °C for 5 min, followed by 28 cycles of 95 °C for 45 s, 55 °C for 50 s, and 72 °C for 45 s with a final extension at 72 °C for 10 min). High-throughput paired-end sequencing was performed on Illumina Miseq PE300 platform after PCR products’ purification (Agencourt AMPure XP Kit). The raw data were first split into samples according to the barcode sequence by the QIIME1 (v1.8.0) software. Data shorter than 230 bp or with a quality score ≤ 20 were screened and removed with Vsearch (v2.7.1) and Pear (v0.9.6). Then, the data were further trimmed with Illumina Analysis Pipeline (v2.6). When splicing, the minimum overlap was set to 10 bp, and the mismatch rate was 0.1. The paired-end sequences were clustered into operational taxonomic units (OTUs), and taxonomic classification was performed with Vsearch (v2.7.1) and Ribosomal Database Project (RDP) Classifier tool based on multiple databases including RDP (v16), SILVA (v128), and Greengenes (vRelease13.5) [86].

### UHPLC-Q-Exactive Orbitrap MS analysis

Metabolites were separated and detected using electrospray ionization mass spectrometry operated in positive mode. A QC sample was obtained by mixing equal amounts of each sample. All samples were filtered, and the supernatant was collected for UPLC-Orbitrap MS analysis. To validate the precision and stability of the instrument system and the analysis method, the QC sample was repeatedly analyzed for five times before sample sequencing. During the analysis, all samples were randomly cross-injected. One solvent blank and one QC sample were added after every 10 tested samples for cleanness and stability [87, 88]. Metabolomics analysis was performed on a Thermo Scientific Vanquish UHPLC coupled to a Q Exactive Orbitrap MS system equipped with an electrospray ionization source. Calibration on positive ions mode was carried out with a standard solution which consists of M/Z195 caffeine, M/Z524 MRFA, and M/Z1021-1921 polymer; calibration on negative ions mode was carried out with a standard solution which consists of M/Z265 sodium dodecyl sulfonate, M/Z514 sodium iodide iodate, and M/Z1079-1979 polymer. The parameters were as follows: spray voltage, 3.5 kV; sheath gas pressure, 35 arb; auxiliary gas pressure, 10 arb; capillary temp, 300 °C; ion source temp, 350 °C; scan modes, MS (FullScan, m/z 100-1200) and data-dependent acquisition MS2 (resolution 17,500, normalized collision energy 35 eV, stepped normalized collision energy 30 and 40 eV); and scan range, m/z 80–1200. To investigate the metabolic shifts under different diet patterns, non-targeted LC-MS was applied and the signal responses were collected under ESI+ mode. Taking polar and minor polar components into consideration, ACQUITY UHPLC BEH C18 column (1.7 μm 2.1 mm × 100 mm) and ACQUITY UHPLC HILIC column (1.7 μm 2.1 mm × 100 mm) were applied for a complete detection of MS characteristic data for metabolites profiling. The mobile phases were combined with 0.1% formic acid in water (A) and 0.1% formic acid in acetonitrile (B). The following are the UHPLC HILIC gradient conditions: 0-10 min, 8-30% (A); 10-12 min, 30-30% (A); 12-13 min, 30-8% (A); 13-15 min, 8-8% (A). The following are the UHPLC C18 gradient conditions: 0-10 min, 70-15% (A); 10-11 min, 15-15% (A); 11-12 min, 70-7% (A); 12-15 min, 70-70% (A).

### Data processing and analysis

The raw data analysis was processed using the Sieve software package version 2.1 (Thermo Fisher Scientific Inc., San Jose, CA, USA). Before chemometric analysis, the data from each sample were normalized to the sum of the peak area [89]. By overlapping data of different groups under HILIC and RP-C18 mode respectively, metabolites of intensity > 5e4 were introduced into the SIMCA-P 13.0 software (Umetrics) for PCA and OPLS-DA analyses. OPLS-DA maximize the discrimination between samples assigned to different classes. Variance and predictive ability (R2X, R2Y, Q) were established to evaluate the suitability of the models. In addition, a permutation test (*n* = 200) was performed to validate the models. The scores from each OPLS-DA model were subjected to a CV-ANOVA to test for significance (*p* < 0.05).

Some were identified through a comparison of standard materials, such as l-proline and l-serine, etc. In addition, the Compound Discoverer 2.0 software was used for structure mapping operation based on the database including mzCloud, Chemspider, Kegg, and self-built masslist with accurate MS1 and MS2 data. Automatic mapping analysis was performed with quality control parameters as follows: MS1 accuracy < 5 PPM, HighChemLow + HighRes applied for spectral library search algorithm, cutoff with mapping score > 60, isotope matching and background noise deducting were applied. The unrecognized ions were uploaded to reliable online databases such as the Human Metabolome Database (http://www.hmdb.ca/) (2020-08-01) and Metlin (http://metlin.scripps.edu/) (2020-08-01) for further mapping. The metabolites identified by the above strategies were further identified with manual matching. The obtained differential components were analyzed by MeV (Multi Experiment Viewer, v4.8, TIGR) for hierarchical clustering analysis and *K*-mean clustering analysis. All experimental data were examined by the one-way ANOVA test in the SPSS 22.0 software to calculate the statistical significance. *p* value < 0.05 was considered significant.

The IPA software (2020-08-15) was applied for bio-functions and pathways enrichment analysis based on differential metabolites that detected with HILIC separate mode. LEfSe analysis was performed on the platform of http://huttenhower.sph.harvard.edu/galaxy.

By using the R WGCNA package (version 0.67) (https://cran.r-project.org/package=WGCNA), WGCNA [90] was performed for distinguishing the gut microbiota component clusters which were associated with the vegetarian diet duration and major fecal metabolites. As for the WGCNA procedure, firstly, the co-expression correlation coefficient of each gene was calculated according to the measured gene expression level. Then, the Euclidean distance was used to cluster genes and draw gene tree. The distance of this gene tree was in line with the scale-free network and more in accordance with the natural law.

The constructed gene tree was pruned by dynamic shearing, and the gene modules were obtained by fusing the pruned gene tree, so that the analysis of a large number of genes was changed to a few gene modules (the color blocks on the bottom of Fig. 4A, please note that the blocks were colored without merging the same color into single one). Then, the phenotypic traits concerned in the study were introduced for weighted analysis, the correlation and reliability of all genes in each gene module (a total of 18 color blocks positioned on the left of Fig. 4B) with phenotypic traits (six fecal metabolites and the vegetarian diet duration) were calculated, and the most relevant and significant modules (|correlation coefficient|> 0.25 and *p* value < 0.05) were selected as the core modules, which are detailed in Fig. 5A.

## Supplementary Information

**Additional file 1.** Figure S1. A, B. Volcano plots based on data detected by RP-C18 and HILIC columns. C, D. 200-times permutation test results which illustrate the robustness of the PLS-DA models as no over-fitting was observed (R2=0.748, Q2=-0.575, HILIC mode; R2=0.736, Q2=-0.387, RPLC mode).

**Additional file 2.** Table S1. 70 endogenous compounds identified from fecal sample.

**Additional file 3.** Table S2. WGCNA MICROBIOTA MODULE.

**Additional file 4.** Table S3. WGCNA Module Traits.

## Data Availability

Gene sequencing data are available in the NCBI Sequence Read Archive under accession number PRJNA690694. Metabolic raw data are available in the UCSD Metabolomic Workbench with data trace ID 2809 and 2808.
