# Supplementary material for: Vegetarian diet duration’s influence on women’s gut environment
Source: Genes Nutr. 2021 Oct 2;16:16. doi: 10.1186/s12263-021-00697-1 (PMC8487541; doi:10.1186/s12263-021-00697-1)
Supplement: Supplementary file 1 — Additional file 1. Figure S1. A, B. Volcano plots based on data detected by RP-C18 and HILIC columns. C, D. 200-times permutation test results which illustrate the robustness of the PLS-DA models as no over-fitting was observed (R2=0.748, Q2=-0.575, HILIC mode; R2=0.736, Q2=-0.387, RPLC mode). [file 12263_2021_697_MOESM1_ESM.docx]

**Figure S1. A, B.** Volcano plots based on data detected by RP-C18 and HILIC columns. **C, D.** 200-times permutation test results which illustrate the robustness of the PLS-DA models as no over-fitting was observed (R2=0.748, Q2=-0.575, HILIC mode; R2=0.736, Q2=-0.387, RPLC mode).

Table.S1 70 endogenous compounds identified from fecal sample

See supplementary excel document.
